# Complete Genome Sequence of a Newly Emerging Newcastle Disease Virus

**DOI:** 10.1128/genomeA.00196-13

**Published:** 2013-05-09

**Authors:** Jing-Yu Wang, Wan-Hua Liu, Juan-Juan Ren, Pan Tang, Ning Wu, Hung-Jen Liu

**Affiliations:** College of Veterinary Medicine, Northwest A&F University, Yangling, Chinaa;; Institute of Molecular Biology, National Chung Hsing University, Taichung, Taiwanb;; Agricultural Biotechnology Center, National Chung Hsing University, Taichung, Taiwanc

## Abstract

The complete genome sequence of a newly emerging Newcastle disease virus, isolated in China, was determined. A phylogenetic analysis based on the F gene revealed that the isolate is phylogenetically related to Newcastle disease virus genotype VIId. Sequence analysis indicated that amino acid residue substitutions occur at neutralizing epitopes on the hemagglutinin-neuraminidase (HN) protein.

## GENOME ANNOUNCEMENT

The hemagglutinin-neuraminidase (HN) glycoprotein of Newcastle disease virus (NDV) is a type II membrane protein, existing on the surface of virions. The ectodomain of the HN glycoprotein comprises a membrane-proximal, stalk-like segment supporting a terminal globular domain. The antigenic, receptor recognition, and neuraminidase (NA) active sites all reside in the globular domain (1, 2). The HN glycoprotein mediates the attachment to a sialic acid-containing receptor(s), and the apparently opposing activity of the release of sialic acid from soluble and membrane-associated glycoconjugates is also regulated by its NA activity (3–7).

In this study, an NDV epidemic strain (NDV/Chicken/TC/9/2011) from chicken flocks in Shanxi Province, China, was isolated in 2011. Eight primer pairs used in reverse transcription-PCR (RT-PCR) were designed to amplify the full length of the genome sequence of the isolate. The full length of the genome sequence of NDV is 15,192 bp. The isolate has the amino acid sequence ^112^RRQKR^116^ at the C terminus of the F2 protein and F at residue 117, the N terminus of the F1 protein (8, 9). On the basis of pathogenicity, NDVs have been categorized into velogenic, mesogenic, and lentogenic pathotypes. The isolate was classified as velogenic NDV with a mean death time (MDT) of 38 h and with an intracerebral pathogenicity index (ICPI) of 1.67. A phylogenetic tree based on the F gene sequence indicated that the isolate was phylogenetically related to NDV genotype VIId. Sequence analysis also found that amino acid residue substitutions occur in the F protein of NDV.

Furthermore, we discovered that recent virulent isolates have an HN glycoprotein consisting of 571 amino acid residues. Previous studies indicated that there are at least five antigenic sites related to epitopes on the HN glycoprotein of NDV, including residues 193 to 201 (site 23), residues 345 to 355 (sites 1 and 14), and a C-terminal domain composed of residues 494, 513 to 521, and 569 (sites 12 and 2) (1, 10). The region with the amino acid residues 341 to 355 of the HN glycoprotein was also defined as a linear epitope, and residues 352 to 355 are required for antibody recognition. Compared with the HN glycoprotein sequences of many traditional NDV vaccine strains (LaSota, Clone 30, B1, and V4), the deduced amino acid sequence of the HN glycoprotein of the newly emerging Shanxi NDV isolate revealed many amino acid residue substitutions at neutralizing epitopes on the HN glycoprotein, such as the amino acid residue substitutions E347R, Y350H, G494D, S518N, and S519L. These sites were associated with antibody recognition. Therefore, this divergence further suggests that amino acid residue substitutions might lead to the change of antibody recognition capabilities and types. It can be speculated that immunization failure might be caused by differences in the antigenic sites between the vaccine strains and field strains (9, 11). Understanding the prevalence and variation of newly emerging NDV isolates in this region has great significance for the prevention and control of Newcastle disease in northwestern China.

### Nucleotide sequence accession number.

The full-length sequence of the newly emerging NDV strain NDV/Chicken/TC/9/2011, which was isolated in northwestern China, has been deposited in GenBank under the accession no. KC461214.
